# The effects of autonomous sensory meridian response (ASMR) on mood, attention, heart rate, skin conductance and EEG in healthy young adults

**DOI:** 10.1007/s00221-022-06377-9

**Published:** 2022-05-05

**Authors:** H. J. Engelbregt, K. Brinkman, C. C. E. van Geest, M. Irrmischer, J. B. Deijen

**Affiliations:** 1 Hersencentrum Mental Health Institute, Amsterdam, The Netherlands; 2 Department of Psychiatry and Psychotherapy, Ludwig-Maximilians-University of Munich, Munich, Germany; 3 Faculty of Behavioural and Movement Sciences, Department of Clinical, Neuro- and Developmental Psychology, Section Clinical Neuropsychology, Vrije Universiteit, Amsterdam, The Netherlands

**Keywords:** ASMR, Attention, Flanker task, Heart rate, Skin conductance, EEG

## Abstract

Autonomous sensory meridian response (ASMR) is a warm tingling sensation which is often accompanied by feelings of calmness and relaxation. The present study examined the effects of an ASMR video on mood, attention, heart rate (HR), electrodermal activity (EDA), electroencephalography (EEG) and the interaction with personality factors in 38 young adults (33 females and 5 males). Based on the ASMR-checklist responses of having tingles during watching the ASMR video 15 participants out of 38 were classified as ASMR-experiencers. Mood, attention and personality characteristics were measured by the Profile of Mood States, the Flanker task and HEXACO. EEG, HR and EDA were recorded during the ASMR and control videos. Depressive feelings decreased after watching the ASMR video in individuals experiencing tingles relative to those not experiencing tingles. Furthermore, in all participants, irrespective of experiencing tingles, a decrease of HR during watching the ASMR video was found. In ASMR-experiencers scoring low on Conscientiousness EDA tended to increase and HR tended-relatively to the group not experiencing tingles—to decrease during watching the ASMR video. EEG recordings indicated that watching the ASMR video was associated with decreased alpha power in ASMR-sensitive participants and decreased theta as well as increased beta power in the whole group of participants. The observed ASMR-induced decrease of alpha and theta power and increase of beta power and (only in low conscientious participants) EDA may reflect that, apart from relaxation, ASMR is related to arousal and focused attention.

## Introduction

### General background

Autonomous sensory meridian response (ASMR) is a sensory and emotional phenomenon, characterized by the experience of a ‘tingling’, static-like sensation, which is generally called paresthesia. The tingling can be felt at the scalp, back of the neck, upper spine, or in further bodily areas. The sensation is in response to specific audio -, visual -, or tactile stimuli, or even elicited by intentional attention control. These external (and often social) triggers include whispering, soft-speaking, tapping, scratching, slow and expert hand movements and close personal attention (Barratt and Davis 2015). Despite the scarcity of scientific studies on this phenomenon, ASMR has gained a good deal of popularity amongst new generations and media culture over the last few years. Especially through YouTube videos, where ‘ASMRtists’ try to trigger the response in the viewer through sounds or visual stimulation in this so-called ‘whisper community’ (Andersen 2015). However, it appears that not everyone experiences ASMR and even aversive, annoying, or ‘cringeworthy’ responses have been documented (Barratt et al. 2017; Poerio et al. 2018). In addition, ASMR experiencers have been found to have elevated levels of misophonia (sensitivity to sound, giving rise to minor annoyance to extreme distress) as compared to age and gender matched controls and measured by the Misophonia Questionnaire (MQ) (Janik McErlean and Banissy 2018).

ASMR may also cause a form of frisson, similar yet distinctive from aesthetic chills caused by music or awe-inspiring scenarios (Poerio et al. 2018). Indeed, it must be noted that ASMR and frisson are distinctive phenomena, although ASMR and frisson appear to have similarities such as pleasurable pilomotor activation and/or goosebumps with a common topical distribution in neck, shoulders and head. For instance, ASMR responders more frequently describe the topical (skin-related) response as a ‘tingling’ sensation rather than as goosebumps. In addition, tingling feelings from ASMR are more sustained and subdued in comparison to frisson, while a most notable difference is that the stimuli associated with ASMR are much quieter, more proximate and involve more human whispered speech (Kovacevich and Huron 2019). Additional evidence of a divergence between the constructs was provided by the finding that responses to ASMR stimuli were not related to scores on the frisson subscale (Roberts et al. 2020).

Several suggestions have been made to explain the occurrence of the ASMR-related tingling sensation, including having specific personality traits (Fredborg et al. 2017; Janik McErlean and Banissy 2017) and the level of connectedness of the Default Mode Network (DMN) areas in the brain (Poerio et al. 2018; Smith et al. 2017). Furthermore, the type of trigger and the individual’s surroundings might play a role in whether ASMR is felt or not (Barratt et al. 2017).

### ASMR and mood

Experiencing ASMR seems to bring about a better mood which is characterized as relaxing. For instance, Poerio et al. (2018) did an online experiment in 1002 participants (813 experiencing ASMR). The researchers presented two ASMR videos and a control video (non-ASMR), which all lasted approximately 3 min. Participants who were experiencing ASMR felt more excitement, calmness and less stress and sadness after watching the ASMR video compared to participants who were not sensitive to ASMR. Thus, results suggested that ASMR induces a positive affect like calmness and excitement only in people who are sensitive to ASMR. (Barratt and Davis 2015) conducted an online questionnaire to determine the prevalence of particular features of ASMR as measured by an interactive sliding scale. Of 475 participants, 80% reported that ASMR improved their mood, while 14% felt unsure, and only a remaining 6% of participants reported no alteration of their mood due to ASMR. Depressive mood state in particular appeared to have improved substantially. Notably, half of the participants reported mood improvement in the absence of tingling sensations. Other authors (Ahuja and Ahuja 2019) suggest that the positive emotions that ASMR seems to provoke, can be deemed a placebo effect, mediated by identifiable health care settings, emotional and cognitive engagement with clinicians and empathetic and intimate witnessing. It has also been suggested that ASMR responses are elicited by the knowledge of what types of experiences one should expect. Indeed, the presence of demand characteristics or expectation effects has been found to specifically exist in ASMR naïve participants (Cash et al. 2018). In this particular study naïve participants experienced ASMR when the neutral audio clips were prefaced by leading instructions, while experienced ASMR users only reported experiencing ASMR in response to audio clips that were intended to elicit ASMR.

### ASMR and cognitive performance

In addition to mood, ASMR has been suggested to improve cognitive abilities, such as attentional performance or decision making. In a lab-based study a 15 min ASMR audio clip containing intimate whispering was presented to 109 ASMR individuals and 103 control participants (Wang et al. 2020). ASMR individuals were selected based on their responses on (1) an ASMR checklist (Fredborg et al. 2017) and, (2) the question (‘Have you ever felt ASMR when listening to these kinds of sounds or not?’) after listening to a 3 min ASMR audio clip. With respect to executive function (EF), it was found that ASMR-triggering audio clips impaired set shifting and inhibitory control, but not working memory, in ASMR individuals as compared to control individuals. The authors conclude that these findings are in accordance with results in some resting-state fMRI studies (e.g., Raichle 2015) concerning the activation of the default mode network (DMN) indicating atypical functional connectivities among some EF-related brain networks in ASMR. Notably, in spite of a possible atypical brain network ASMR individuals did not show any deficiency in executive functioning, as set shifting and inhibitory control were only interfered when ASMR was elicited.

### ASMR and brain imaging

Concerning the neural underpinnings of ASMR, one of the first studies made a comparison between fMRI recordings of 11 individuals who were able to experience ASMR with those of 11 matched controls (Smith et al. 2017). The results indicated that the DMN of individuals who experienced ASMR showed less functional connectivity between the frontal lobes and sensory and attentional areas than the control group. In a subsequent fMRI study participants who were self-identified as sensitive to ASMR watched six 4 min videos (three ASMR videos and three control videos) while undergoing an fMRI scan (Smith et al. 2019). The ASMR videos activated brain areas related to sensation, emotion, motor skills and attention, suggesting that ASMR is not confined to sensory or emotional experiences but also related to motor skills and cognitive functioning.

Another study similarly examined which brain areas of ASMR-sensitive individuals are active while watching ASMR videos (Lochte et al. 2018). While undergoing a fMRI scan the participants watched five 7 min ASMR videos and had to press one of the three buttons for a baseline sensation, relaxation or tingling sensation. During tingling sensations brain activation was found in the medial prefrontal cortex, which is associated with self-awareness and social cognition. Besides that, a significant activation in regions associated with reward (nucleus accumbens) and emotional arousal (corpus callosum and Insula/inferior frontal gyrus) was found.

### ASMR and physiological measures

Besides influences on mood and brain activity, ASMR has been linked to physiological effects. Poerio et al. (2018) measured the effects of ASMR on heart rate (HR) and skin conductance level (SCL). Participants who experienced ASMR showed a reduced HR, suggesting that ASMR is related to relaxation. Furthermore, participants who experienced ASMR had a higher SCL after watching the ASMR videos. Skin conductance is an indicator of physiological arousal during emotional, cognitive and physical behavior and decreases with physiological relaxation like sleep or rest (Nagai et al. 2004). The finding that skin conductance level increases after watching the ASMR videos contradicts with the relationship found between ASMR and relaxation. However, Poerio et al. (2018) also found that ASMR induces excitement in individuals who experienced ASMR, suggesting that ASMR may not be only associated with relaxation but also with increased arousal.

In addition to studies on HR and SCL, one study on pupil response found that the tingling sensations experienced in ASMR were accompanied with increases in pupil diameter (Valtakari et al. 2019). These results demonstrate that tingling sensations have a physiological basis and may be considered to be the core of the experience itself.

### ASMR and personality characteristics

Several studies have shown that the ability to experience ASMR is associated with personality characteristics. For example, compared to matched controls individuals experiencing ASMR had higher scores on scales of Openness-to-Experience and lower scores on scales of Conscientiousness (Fredborg et al. 2017; Janik McErlean and Banissy 2017), higher scores on Neuroticism and lower scores on scales of Extraversion and Agreeableness (Fredborg et al. 2017). The occurrence of frisson and the experience of ASMR are both activated by focusing on triggering stimuli, while individuals with high openness-to-experience might be more sensitive or receptive to these triggers.

### ASMR and electroencephalography

With respect to ASMR and brain activity, this association has mostly been studied by fMRI recordings. Notably, one recent study examined ASMR by means of EEG recordings (Fredborg et al. 2021). Relative to the control group the ASMR experience elicited by video—and auditory stimuli was reflected by increased alpha activity in frontal and parietal brain regions. Increased frontal alpha activity was also detected throughout audio trials when brain activity during ASMR tingles was compared to pre-tingle activity.

Although less pronounced, increased activity of sensorimotor rhythm (12.5–15 Hz) occurred for audio trials when comparing ASMR to control participants and gamma waves (> 30 Hz) when comparing pre-tingle to post-tingle activity in ASMR participants. Higher levels of sensorimotor rhythm activity were also seen over the motor and somatosensory cortices during ASMR participants’ responses to ASMR-relevant audio files as compared to ASMR-irrelevant audio files. The authors conclude that increases of alpha, sensorimotor rhythm and gamma wave activity might reflect attentional and sensorimotor processes during ASMR in ASMR-sensitive individuals.

### ASMR and meditation

To examine the relationship between ASMR and mindfulness, Roberts et al. (2021) measured ASMR propensity in 318 participants (139 males, 169 females) between 18 and 72 years recruited from Facebook and Reddit with the Autonomous Sensory Meridian Response Scale (ASMR-15), which includes the subscales Affect, Altered Consciousness, Sensation and Relaxation. Mindfulness was measured by the Mindful Attention and Awareness Scale (MAAS), a 15-item measure of awareness and attention in every day experiences. ASMR propensity appeared to be weakly negatively related to trait mindfulness, particularly the Altered Consciousness dimensions of ASMR experiences. These findings can be explained by the notion that absorption tendency underlying ASMR experiences appears incompatible with trait mindfulness.

In a preceding study, a group of ASMR experiencers was compared with an age and gender matched control group on measures of absorption, flow and mindfulness. Results indicated no difference between ASMR-experiencers and control group concerning mindfulness, as measured by the MAAS. In addition, no correlation was found between ASMR characteristics determined with a self-designed ASMR questionnaire and scores on the MAAS. Notably, increased absorption was found in the ASMR group while no association between absorption and mindfulness was found. These results show that ASMR and mindfulness are different phenomena (Janik McErlean and Osborne-Ford 2020).

In contrast to the above evidence of being distinct phenomena, ASMR seems to have similarities with meditation techniques. Both mindfulness meditation and ASMR can lead to a feeling of relaxation that enhances subjective well-being (Barratt and Davis 2015). In a study reporting a relationship between ASMR and mindfulness the scores of ASMR experiencers on the Toronto Mindfulness Scale (TMS), the MAAS and a ASMR questionnaire were compared with those of age- and sex-matched control participants (Fredborg et al. 2018). ASMR experiencers scored higher on the MAAS and the Curiosity subscale of the TMS. Results support the hypothesis that ASMR is related to mindfulness, in that way that the ASMR-induced sensory-emotional experiences are partially explained by mindfulness-like characteristics.

Several studies showed a relationship between forms of meditation (i.e., mindfulness, yoga), relaxation, anxiety and stress (Grossman et al. 2004; Smith et al. 2007). Cahn and Polich (2006) reviewed several EEG-studies on meditation and concluded that the power of alpha and theta frequency bands increases during meditation. Furthermore, Takahashi et al. (2005) recorded EEG activity in young healthy adults during a period of rest and while the participants performed a meditation task. The researchers found an increase of the fast theta (6–8 Hz) and the slow alpha frequency bands (8–10 Hz) during meditation, particularly in the frontal area.

### Present study

The aim of the current study was to examine the effects of ASMR stimulation on mood, attention, heart rate, skin conductance level and EEG and its relationship with personality characteristics. By applying a multimodal approach we aimed to replicate previous findings in one group of participants. In addition, the present objective was to examine the interrelationship between ASMR sensitivity, physiological measures and personality factors. As ASMR is associated with a reduced HR and high conscientiousness with lower cardiovascular reactivity (Merecz et al. 1999), conscientiousness was expected to be the most likely mediating personality factor.

We hypothesized that ASMR improves mood and attention. To cover most of the above mentioned findings in the ASMR (Fredborg et al. 2021) and meditation studies of an increase of theta, alpha, sensorimotor rhythm and gamma in frontal and/or parietal brain regions, we hypothesized that ASMR changes the brain activity by inducing an increase in theta, alpha and/or beta power in the frontal and parietal regions. We did not examine gamma power as changes in this frequency band were less pronounced and mainly seen within the ASMR participants group.

Furthermore, in line with former findings (Poerio et al. 2018) we expected that ASMR decreases heart rate and increases electrodermal activity. Finally, we hypothesized that individuals who score lower on extraversion and conscientiousness and those who score higher on neuroticism, agreeableness and openness are more sensitive to ASMR.

## Methods

### Participants

The study sample consisted of 38 participants. All participants were Dutch undergraduate Psychology students at the Vrije Universiteit Amsterdam, ranging from ages 17 to 35 years, with a mean age of 20.42 years (SD = 2.85). According to the inclusion criteria participants should only be healthy and aged between 17 and 35 years. No selection was made based on self-described ASMR experience nor were participants explicitly informed of the ASMR content of the study. The study included 33 female and 5 male participants. According to their own knowledge they were mentally and physically healthy. The participants were recruited by means of vu.sona-systems.com. This is a subject pool where students of the Vrije Universiteit may indicate to participate in research, which is rewarded by credits.

## Materials

### ASMR questionnaire and video

An adapted ASMR-checklist of Barratt and Davis (Barratt and Davis 2015) was used to indicate if participants experienced tingles or not. A selection of 15 items of the questionnaire was made and these items were translated into the Dutch language. The translated items concern 6 demographic questions (e.g. gender, age, use of medication, chronic pain or illness, frequency of looking at ASMR videos), 2 items concerning the occurrence of tingles and the specific body areas, and 7 items concerning the occurrence of flow. The original question ‘Do you feel a tingling sensation when watching ASMR videos?’ was rephrased as: ‘Did you feel a tingling sensation while watching the video (yes/no)?’ We included one additional item on the pleasantness of the videos. With respect to the original and adapted version no psychometric properties are available.

The ASMR and control video were the same standard ASMR and control (non-ASMR) video as used in study 2 by Poerio et al. (2018). The ASMR video lasted 3.20 min and included a soft voice of a female who is folding towels. The video clips were taken from YouTube (https://www.youtube.com/watch?v=t0tSc-1qdKA). The control video lasted 3.04 min and included a neutral voice of a male, explaining how to make fresh pasta (https://www.youtube.com/watch?v=H8pAFgky-Xo). The video clips were presented on an iPad. To avoid disturbing the EEG recordings participants did not wear headphones but listened to the sounds from the iPad speaker. The order of the presentation of the videos was randomized.

### Mood

Mood was measured by the Profile of Mood States (POMS) (McNair et al. 1971). In the current study, we used a shortened version (Wald and Mellenbergh 1990). The POMS exists of 5 subscales: Anger, Depression, Tension, Fatigue and Vigor.

These five scales are operationalized in 32 adjectives which are rated on a 5-point Likert scale (ranging from 0 ‘Not at all’ to 4 ‘Very much’). Examples of adjectives are: ‘sad’ or ‘tired’. After adding 1 point to each rating, the ratings 1–5 of the items of a subscale are summed. The scales are composed of a different number of items, i.e., Anger contains 7 items (range of scores 7–35), Depression 8 items (range of scores 8–40), Tension 6 items (range of scores 6–30), Fatigue 6 items (range of scores 6 to 30) and Vigor 5 items (range of scores 5–25).The higher the scores on a scale are, the larger the emotional disturbance, except for vigor (higher score means more vigor). The Dutch POMS questionnaire is a reliable measurement with a Cronbach alpha for different scales, varying between 0.85 and 0.95 (de Groot 1992; Wald and Mellenbergh 1990; Wicherts and Vorst 2004).

### Attention

Attention was measured by the Eriksen Flanker task (Eriksen and Eriksen 1974). The Flanker task consists of two trial types, with either congruent or incongruent visual stimuli. The congruent trial is a horizontally arranged array of arrows presented in the same direction (e.g., <  <  <  <  < or >  >  >  > >). The incongruent trial has a similar array of arrows, but the middle arrow, the target, was displayed in the opposite direction (e.g., <  <  >  <  < or >  >  <  > >). Participants were asked to respond to the direction of the target arrow within the array of arrows by pressing the corresponding right or left button (e.g., pressing the right button marked “R” for <  <  >  <  < ; pressing the left button marked “L” for >  >  <  > >) as quickly and accurately as possible. A fixation cross was displayed on the center of the computer screen for 1000, 1500 or 2000 ms (fully random), followed by an imperative stimulus with a 2500 ms presentation time. The interstimulus interval (ISI) was 100 ms, and failure to respond within 2500 ms or pressing the wrong button was considered an incorrect response. The size of the fixation cross was 2 × 2 cm. The size of the individual arrows were 2, 3 × 22 cm with 06 cm spacing between the arrows. Both the fixation cross and the arrows were set in the middle of the screen in a 150 × 2,4 cm frame on a 197 × 147 cm iPad screen. A total of 60 response trials were presented in one block, in which the order of congruent and incongruent trials was randomized with the same probability. The total task duration was approximately 3.5 min. The accuracy percentage and reaction times of correct responses on incongruent trials were identified as metrics of behavioral cognitive performance, when the accuracy was at least 80%. The reaction time (RT) and the number of correct responses were taken as output variables.

### Personality

Personality was measured by the HEXACO-Simplified Personality Inventory (HEXACO-SPI). The scale has an adequate factor structure, reliable domain scales (mean rXX = 0.79), more than adequate convergent validity and highly convergent construct validities. The HEXACO model is an extended version of the Big Five personality model (John et al. 1991), with Honesty-Humility as additional factor (de Vries and Born 2013). Ludeke et al. (2019) reported meta-analytic correlations between HEXACO and Big Five Inventory (BFI) scales, i.e. Extraversion (*r* = 0.81), Conscientiousness (*r* = 0.76), Openness to experience (*r* = 0.74), Emotionality/Neuroticism (*r* = 0.52) and Agreeableness (*r* = 0.37). The self-report HEXACO-SPI consists of 104 items, like ‘My room is always cleaned up’. Participants indicate to what extent these statements are correct. Response options are 1 = totally disagree, 2 = disagree, 3 = neutral, 4 = agree, 5 = totally agree. Domains of the HEXACO-SPI are Honesty-Humility, Emotionality, Extraversion, Agreeableness, Conscientiousness, Openness to experience. For the current study we analyzed the scores on Emotionality, Extraversion, Agreeableness, Conscientiousness and Openness to experience.

### Heart rate and electrodermal activity

Heart rate (HR) in beats per minute (bpm) and electrodermal activity (EDA) in microSiemens (µS) were measured by the VU-AMS (Vrije Universiteit-Ambulatory Monitoring System; http://www.vu-ams.nl). This system was developed at the Vrije Universiteit Amsterdam. For EDA recording we used electrodes from Biopac (Skin Resistance Trans, TP–TSD203) combined with electrode gel, isotonic, 4OZ–GEL101. To measure the HR we used Kendall ARBO H98SG ECG electrodes. Two skin conductance electrodes were placed on the thenar eminence of the left hand and forearm, because the instruction manual recommends this configuration to attain optimal signal quality. Two HR electrodes were placed on the left forearm and under the right clavicle. The mean HR and EDA during the two videos were taken as output variables. The ECG had a sampling rate of 1000 Hz and HR was calculated based on time (in ms) between successive R waves (R-R intervals) on the ECG to calculate average beats per minute. R peaks are automatically detected by the VU-AMS. Data were scored using the Data Analysis and Management Software (DAMS; http://www.vu-ams.nl/vu-ams/software/). The average skin conductance, i.e. the average raw EDA signal, was used in this study. Skin conductance was sampled at a rate of 10 Hz with a signal range between 0 and 95 μS. The signal was filtered both in forward and reverse direction with a low pass filter with a cut off frequency of 2 Hz. Data processing from electrodermal signals was computationally performed with the DAMS. Clipping levels were automatically detected and removed from further analysis.

### Electroencephalography (EEG)

#### EEG recording

EEG was recorded by means of 19-channel electrode caps with international 10–20 electrodes placement (Jasper 1958) on a 32-channel Deymed system (sampling rate 1024 Hz downsampled to 128 Hz, Notch filter 50/60 Hz, anti-aliasing filter 50 Hz, Butterworth filter 0.1–100 Hz). We applied the electrodes with ECI electrode gel. Electrode skin impedance was kept below 8 kΩ. An electrode at Fpz served as ground electrode, while electrodes on the left and right earlobes served as offline linked-ear (LE) reference. The EEG system was connected to a portable computer. EEG activity was analyzed in absolute power, using the Fourier Transformation, and reported in microvolts squared (Thatcher 2008).

#### EEG processing

Artifact-free EEG data were selected by an EEG expert after screening for seizure activity and/or abnormal EEG patterns. Data files were screened for eye blinks, eye-movement in vertical and lateral ways, technical flaws and distortion by frontal and temporally located muscle contractions. After visual inspection of the EEG data the EEG expert applied the program Persyst 14 (Persyst Development Corporation, San Diego) with the built in tool for spike analysis. The Persyst spike algorithm allows the detector to be extremely sensitive while maintaining a low false positive rate, and appeared to perform similar to human EEG readers. A detailed description of the algorithm and comparison with the performance of human EEG readers can be found in Scheuer et al. (2017). For artifact rejection, the automated selection tool of the program NeuroGuide (V3.0.0.1) was used. For ocular artifact rejection electrodes Fp1 and Fp2 were used. Default for eye movement and drowsiness selection is ‘high’ which is the most sensitive setting and 1.5 standard deviations threshold for the Amplitude Multiplier. The *Z* Score of 1.5 standard-deviations means that if at least one second of successive instantaneous Z Scores are equal to or less than 1.5 standard deviations then a selection is made (Applied Neuroscience 2018). The EEG was recorded during the presentation of the videos. Data of individual EEG recordings were included only when there was a minimum of 20 s artifact free data.

For the current study we selected the alpha (8.0–12.0 Hz), theta (4.0–8.0 Hz) and—to include sensorimotor rhythm-beta (12.0–25.0 Hz) frequency bands at channel F7, F8, P3, P4, T5 and T6. As electrodes T5 and T6 are also called parietal-temporal electrodes and have been renamed in the higher-resolution nomenclature (Modified Combinatorial Nomenclature; MCN) P7 and P8 (Oostenveld and Praamstra 2001), we selected these electrode locations to additionally cover parietal measurements.

#### Procedure

The experiment took place in a quiet room. Participants sat in a chair facing a plain white wall. At the start of the experiment the participants received information and signed an informed consent. After participants had completed the HEXACO questionnaire, the HR and SCL electrodes were attached, followed by the EEG cap. The recording of the VU-AMS and EEG amplifier were started and then a period of 3 min’ rest followed. Participants had to look forward at the wall and sit quiet and relaxed. After the rest period participants started with the Flanker task. When they had finished the task, the EEG amplifier was started again and the first video was presented. After watching the first video, the participants performed the Flanker Task again and they completed the ASMR and POMS questionnaires. Then the second video was presented. After watching the second video the participants performed the Flanker task again and completed the ASMR and POMS questionnaires. To minimize ASMR expectancy effects as cited above (Cash et al. 2018), participants were not informed of the ASMR content of the study. Participants were only told that the aim of the study was to examine the effects of different types of videos on mood, attention, heart rate, skin conductance and EEG. After all participants had watched the same two videos in a counterbalanced order they were assigned to the ASMR and non-ASMR group after completing the experiment. This assignment took place according to their response on the ASMR question whether or not they experienced tingling sensations. This procedure is similar to that applied in a former study (Valtakari et al. 2019).

Ethical approval was obtained from the Scientific and Ethical Review Board of the Faculty of Behavioral and Movement Sciences of the Vrije Universiteit.

### Statistical analysis

Based on the responses to the question of the ASMR-checklist of having had tingles during watching the ASMR video 15 participants out of 38 were classified as ASMR-experiencers. Prior to testing the hypotheses, the assumption of normality of the dependent variables was tested by the Kolmogorov–Smirnov Test. Normality could not be assumed for the scores of the POMS and the correct responses on the Flanker task. Furthermore, the scale of the POMS was ordinally distributed. In addition, normality could not be assumed for the difference scores of the POMS. Therefore, non-parametric tests were applied on difference scores of POMS and correct responses on the Flanker task. To simulate mixed ANOVA the difference scores were calculated by subtracting the scores obtained after the control video from those obtained after the ASMR video. We used a Wilcoxon Signed Rank test to analyze the main effect for Condition (ASMR/control) and a Mann–Whitney *U* test to analyze the relationship between Group (tingles/no tingles) and the difference scores. The effect of Group and Condition on RT of the Flanker task, HR, SCL and EEG were tested by mixed ANOVA. For the separate mixed ANOVAs in groups with high and low conscientiousness two groups were created by the SPSS procedure ‘visual binning’. We used the visual binning option ‘equal percentiles’ which resulted in one group consisting of participants with high scores and one group with low scores. As tension is known to be associated with a higher cardiovascular reactivity (Brouwer and Hogervorst 2014), the relationship between HR and POMS Tension scores was examined by Spearman correlations. The relationship between Group and Personality was examined with Pearson point biserial correlations between Group (tingles/no tingles) and scores on HEXACO scales Emotionality, Extraversion, Agreeableness, Conscientiousness and Openness to experience. To ascertain that data from our 38 participants provided enough power to detect the hypothesized effects, we conducted a power analysis for ANOVA (within-between interaction) using the program G* power 3.1.9.4 (Faul et al. 2007). After applying an effect size η2 = 0.3 (similar to *f* = 0.65, correlation = 0.2, 2 measurements (ASMR/control) and 2 groups (tingles/no tingles or high/low personality trait), the obtained power was 0.89 for sample size = 12. With respect to the relationship between Group and Personality we conducted a power analysis for Pearson point biserial correlation using the program G* power. After applying a medium effect size *r* = 0.4 (McLeod 2019) the obtained power was 0.83 for sample size = 37.

We controlled for multiple comparisons of the frontal and temporal electrodes by applying Benjamini–Hochberg. An FDR of 0.05 can be too low in experiments of an exploratory nature (McDonald 2009). As EEG recordings in ASMR research are scarce and can thus be considered to be quite exploratory, we applied Benjamini–Hochberg with an FDR of 0.2. The choice of a higher FDR can avoid missing important results (McDonald 2009). FDR can be applied in smaller studies and has the advantage to increase power when analyzing multiple tests. The practical implications and benefits of applying an FDR level of 0.2 has been illustrated in real examples (Glickman et al. 2014). We applied this procedure separately for 12 frontal, 12 parietal and 12 temporal tests (6 main plus 6 interaction effects).

The hypotheses for mood, attention, HR, SCL, personality and EEG were tested two-tailed. The level of significance was set as *p* < 0.05. Data were analyzed by using SPSS Statistics version 24.0.

## Results

Independent t tests and chi square tests indicated that age (*T*(36) = 1.71, *p* = 0.096) and gender (*Χ*^2^(1) = 2.09, *p* = 0.15) were not different between ASMR groups. To the question ‘how often do you look at ASMR videos?’ most participants answered ‘never’ (ASMR group: never: 10, sometimes: 2, often 3; non-ASMR group: never: 17, sometimes: 5, often: 1). Notably, participants who indicated not to understand the abbreviation ASMR were verbally informed of its meaning. In addition, chi square tests indicated that the number of reported tingles were not significantly different between the group that first watched the control video and the group that first watched the ASMR video (*Χ*^2^(1) = 0.99, *p* = 0.32). As the number of participants reporting tingles (*n* = 6) in the group that first watched the control video was even smaller than that (*n* = 9) of the other group, the ASMR questionnaire presented after the control video seems not to induce ASMR expectancy effects.

**Fig. 1:**
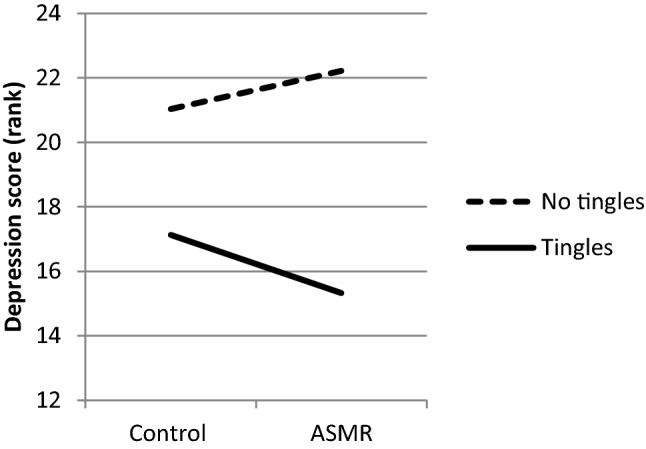
Mean ranks for the Depression scores in the Tingles (*n* = 15) and No-tingles (*n* = 23) group under the control and the ASMR condition

The first hypothesis, ASMR improves mood, was tested by comparing difference scores (ASMR minus control) between Groups (tingles/no tingles) with a Mann–Whitney test. A significant effect was found for the difference scores of Depression (*U* = 100, *z* = − 2.318, *p* = 0.03). Participants who experience tingles showed a smaller change (mean rank = 14.67) in Depression than those who did not experience tingles (mean rank = 22.65). This result is shown in Fig. 1. The scores of Depression decreased under the ASMR condition as compared to the control condition in the ‘tingles group’ relative to the ‘no tingles group’. Mann–Whitney test did not indicate a significant difference in Depression score between Tingles and No-tingles group under the control condition (*p* > 0.05), but only under the ASMR condition (*p* = 0.043). However, this difference was not significant after Bonferroni adjustment for multiple tests. Moreover, Wilcoxon signed-rank tests did not indicate a significant change of Depression scores under the ASMR condition as compared to the control condition in the tingles group nor in the no tingles group (*p* > 0.05). There were no significant differences between Groups for Anger, Fatigue, Vigor and Tension (*p* > 0.05). The main effect of Condition (ASMR/control) on mood was tested with a Wilcoxon Signed Rank test. There was no significant effect of Condition on any of the five mood scales (*p* > 0.05). See Table 1 for the descriptive statistics.

**Table 1:** Mean and difference (ASMR minus Control) scores (SD) of the POMS subscales for Groups (tingles *n* = 15; no tingles *n* = 23) under the control and the ASMR condition

	Control		ASMR		Difference score	
	Tingles	No tingles	Tingles	No tingles	Tingles	No tingles
Depression^a^	9.27 (2.82)	10.04 (2.93)	8.93 (2.34)	10.61 (3.26)	− 0.33 (0.90)	0.57 (2.92)
Anger	8.33 (3,27)	8.61 (3.47)	7.93 (1.79)	9.70 (3.80)	− 0.40 (3.02)	1.09 (4.38)
Fatigue	11.00 (5.07)	10.57 (3.74)	10.47 (4.79)	11.04 (4.27)	− 0.53 (2.39)	0.48 (2.25)
Vigor	12.87 (3.54)	13.52(4.71)	13.47 (4.64)	12.61 (4.44)	0.60 (3.13)	− 0.91 (4.48)
Tension	8.73 (3.11)	8.70 (3.36)	8.87 (3.87)	8.48 (2.81)	0.13 (2.10)	− 0.22 (3.18)

^a^*p* = .030

With respect to Attention, a non-parametric Mann–Whitney test did not show a significant difference between Groups (tingles/no tingles) for the difference scores of the number of correct responses of the Flanker task (*p* > 0.05). This means that there was no difference between the groups in change of correct responses under ASMR and control condition. In addition, disregarding Group, a Wilcoxon Signed Rank indicated no significant effect of Condition for the number of correct responses on the Flanker task (*p* > 0.05).

To test the interaction effect of Group (tingles/no tingles) and Condition (ASMR/control) on RT of the Flanker task mixed ANOVA was applied with Group as between subjects factor and Condition as repeated measures factor. No significant interaction was found between Group and Condition (*p* > 0.05). Besides that, there was no significant main effect of Condition on RT (*p* > 0.05). See Table 2 for the descriptive statistics.

**Table 2:** Mean scores (SD) for Groups (tingles *n* = 15; no tingles *n* = 23) on the number of correct responses and RT (sec) of the Flanker task under the control and the ASMR condition

	Control		ASMR	
	Tingles	No tingles	Tingles	No tingles
Correct responses	59.60 (0.91)	59.74 (0.62)	59.67 (0.82)	59.91 (0.29)
Reaction time	0.58 (0.10)	0.60 (0.08)	0.58 (0.01)	0.61 (0.08)

**Fig. 2:**
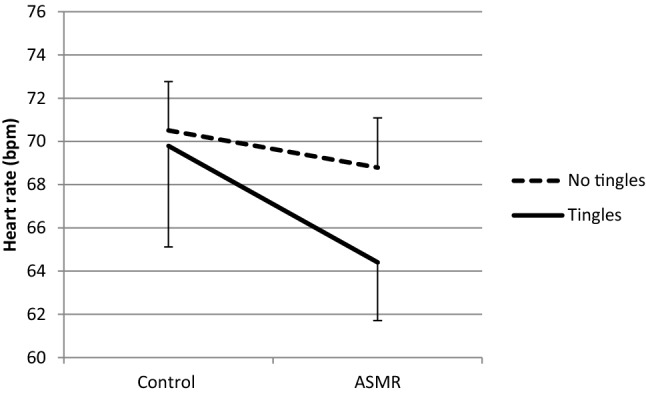
Mean heart rate + SE (bpm) in the Tingles (*n* = 7) and No-tingles (*n* = 14) group within the ‘low conscientiousness’ group under the control and the ASMR condition

**Fig. 3:**
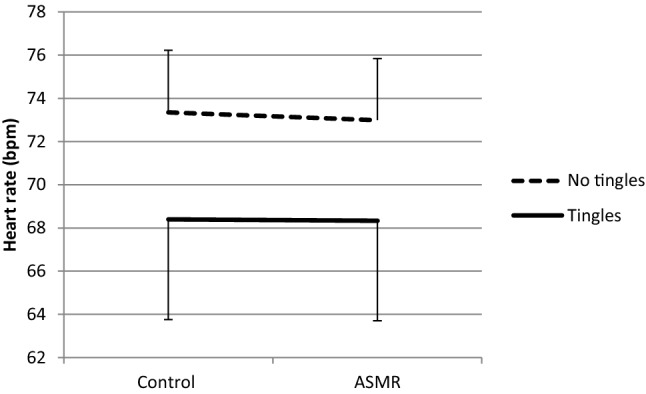
Mean heart rate + SE (bpm) in the Tingles (*n* = 7) and No-tingles (*n* = 8) group within the ‘high conscientiousness’ group under the control and the ASMR condition

The effect of ASMR on HR was tested with mixed ANOVA with Group (tingle/no tingle) as between subjects factor and Condition as repeated measures factor. With respect to the HR analyses data from one participant was excluded from data analysis due to noise in the recordings. Neither significant interaction was found between Group and Condition (*F*(1, 35) = 1.18, *p* = 0.284, *η*^2^ = 0.03, nor a significant main effect of Group (*F*(1, 35) = 0.007, *p* = 0.934, *η*^2^ = 0.00. However, a significant main effect was found of Condition on HR, *F*(1, 35) = 9.85, *p* = 0.003, *η*^2^ = 0.22. The HR under the ASMR condition (68.81 bpm, SD = 8.98) was lower than that under the control condition (70.71 bpm, SD = 9.60). Spearman’s rank correlation indicated a trend of a positive relationship between HR and Tension scores for all participants only under the ASMR condition (*r*_s_ = 0.31, *p* = 0.06, *N* = 36). In addition, mixed ANOVAs with Group (tingles/no tingles) and Condition were performed separately for high and low Conscientiousness. A marginal significant interaction between Group and Condition was found for low Conscientiousness, *F*(1, 19) = 3.32, *p* = 0.084, *η*^2^ = 0.15 (Figs. 2, 3).

**Fig. 4:**
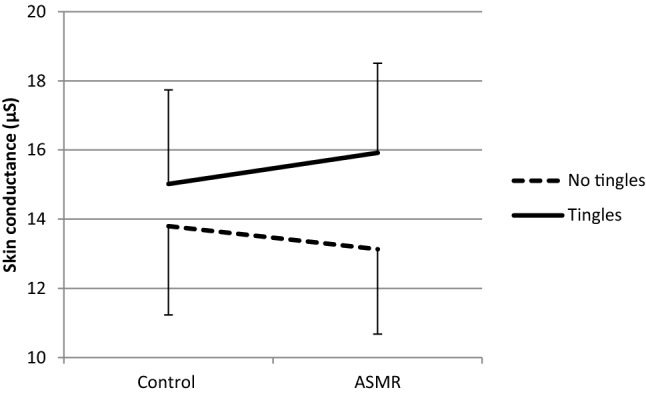
Mean skin conductance + SE (μS) in the Tingles (*n* = 7) and No-tingles (*n* = 14) group within the ‘low conscientiousness’ group under the control and the ASMR condition

**Fig. 5:**
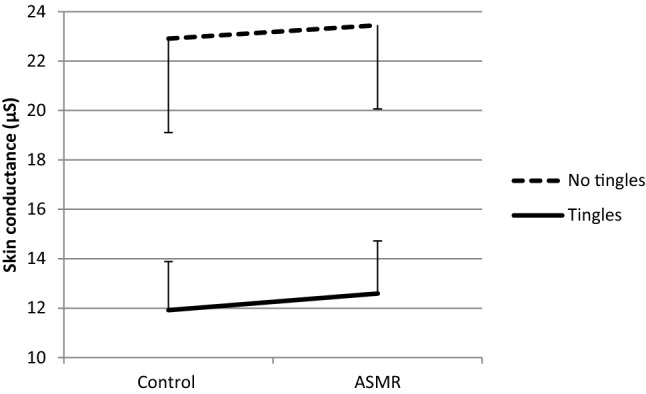
Mean skin conductance + SE (μS) in the Tingles (*n* = 7) and No-tingles (*n* = 8) group within the ‘high conscientiousness’ group under the control and the ASMR condition

With respect to EDA, mixed ANOVA indicated no significant interaction between Group and Condition (*F*(1, 35) = 1.14, *p* = 0.29, *η*^2^ = 0.03). Nor any significant main effect of Group (*F*(1, 35) = 1.09, *p* = 0.30, *η*^2^ = 0.03 or Condition (*F*(1, 35) = 0.27, *p* = 0.61, *η*^2^ = 0.01 on SCL was found. However, mixed ANOVA with Group (tingles/no tingles) and Condition performed separately for high and low Conscientiousness indicated a significant interaction between Group and Condition for low Conscientiousness, *F*(1, 19) = 5.05, *p* = 0.037, *η*2 = 0.21. Paired t tests indicated a marginal significant increase in average EDA under the ASMR condition as compared to the control condition in the tingles group (*p* = 0.077) while no (marginal) significant change was seen in the no tingles group (*p* = 0.95). These results are shown in (Figs. 4, 5). See (Table 3) for the descriptive statistics of HR and EDA.

**Table 3:** Mean heart rate (bpm) and skin conductance (µS) for Groups (tingles *n* = 14; no tingles *n* = 23) under the control and the ASMR condition

	Control		ASMR	
	Tingles	No tingles	Tingles	No tingles
Heart rate^a^	71.32 (10.73)	70.34 (9.08)	68.53 (9.18)	68.99 (9.07)
Skin conductance	13.35 (6.07)	16.99 (10.84)	13.95 (6.16)	16.78 (10.49)

^a^*p* = .003 main effect Condition (Control/ASMR)

**Fig. 6:**
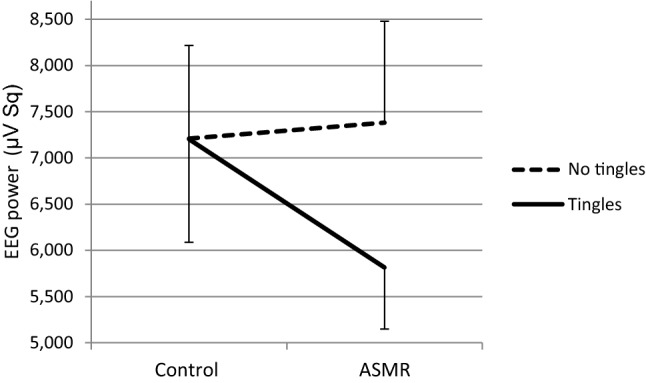
Mean EEG power + SE (µV Sq) in the Tingles (*n* = 13) and No-tingles (*n* = 19) group for the alpha frequency band at channel T6 under the control and the ASMR condition

**Fig. 7:**
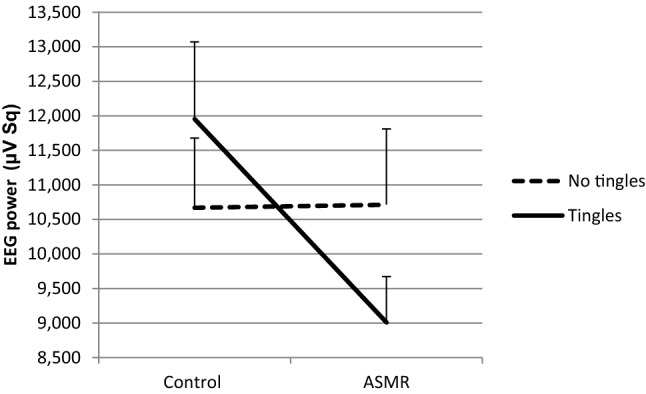
Mean EEG power + SE (µV Sq) in the Tingles (*n* = 13) and No-tingles (*n* = 19) for the alpha frequency band at channel P4 under the control and the ASMR condition

The effect of ASMR on the power of the EEG frequency bands (alpha, theta, beta), was tested with mixed ANOVA. With respect to the EEG analyses data from 6 participants were excluded from data analysis due to noise in the recordings. A (marginal) significant interaction effect of Group (tingles/no tingles) and Condition (ASMR/control) was found for the alpha frequency bands at channel T6, *F*(1, 30) = 4.431, *p* = 0.044, *η*^2^ = 0.13 and at channel P4, *F*(1, 30) = 3.742, *p* = 0.063, *η*^2^ = 0.11. Figure 6 shows a significant decrease (*t*(12) = 2.322, *p* = 0.039) of the power of alpha at channel T6 and Fig. 7 a marginal significant decrease (*t*(12) = 2.055, *p* = 0.062) of the power of alpha at channel P4 in participants who experience tingles as compared to the no tingles group (*p* > 0.05). However, no significant main effect was found for Condition on any channel of the alpha frequency band (*p* > 0.05).

**Fig. 8:**
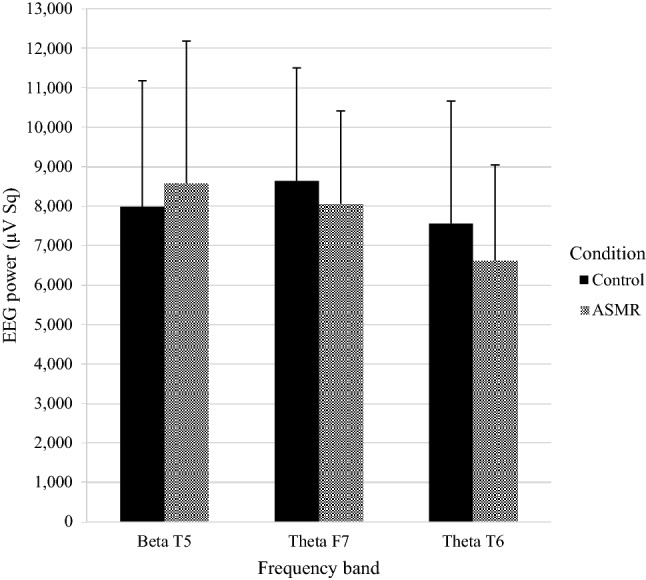
Mean EEG power + SD (µV Sq) for the beta frequency band at channel T5 and theta frequency band at channel F7 and T6 under the control and the ASMR condition (*n* = 32)

A significant main effect of Condition on the beta frequency band was found at channel T5, *F*(1, 30) = 4.510, *p* = 0.042, *η*^*2*^ = 0.13. Figure 8 shows a larger beta power at channel T5 for the ASMR condition compared to the control condition. There was no significant interaction between Group and Condition (*p* > 0.05).

Finally, a significant main effect of Condition was found on the theta frequency band at channel F7, *F*(1, 30) = 6.662, *p* = 0.015, *η*^2^ = 0.18 and channel T6, *F*(1, 30) = 16.554, *p* < 0.001, *η*^2^ = 0.36. Figure 8 shows the smaller theta power at channel F7 and T6 for the ASMR condition compared to the control condition. No significant interaction between Group and Condition was found (*p* > 0.05). All above mentioned results remained significant after controlling for the FDR by the Benjamini–Hochberg procedure. No significant main effects of Condition were found for other channels of the three frequency bands (*p* > 0.05). See Table 4 for the descriptive statistics.

**Table 4:** Mean EEG power (µV Sq) of the alpha, beta and theta frequency bands under the control and the ASMR condition for both groups (tingles *n* = 13; no tingles *n* = 19)*

	Control				ASMR			
	Tingles		No tingles		Tingles		No tingles	
	M	SD	M	SD	M	SD	M	SD
Alpha F7	5.038	2.204	5.041	2.107	4.838	1.910	5.492	2.813
Alpha F8	5.209	1.902	5.323	2.519	4.836	1.600	5.073	2.087
Alpha P3	8.971	4.558	10.406	5.945	9.437	5.437	11.968	9.355
Alpha P4	11.953	8.450	10.670	6.882	9.008	4.692	10.713	7.484
Alpha T5	5.561	2.551	6.254	3.639	5.899	2.853	7.505	5.859
Alpha T6^b^	**7.202**	4.020	**7.209**	4.394	**5.815**	2.396	**7.381**	4.787
Beta F7	8.064	3.614	10.454	7.071	7.692	2.827	7.692	5.875
Beta F8	9.526	4.943	9.885	5.462	9.439	5.289	9.523	3.772
Beta P3	9.787	4.376	9.876	4.367	10.197	3.637	10.158	4.526
Beta P4	10.429	3.751	9.906	4.129	10.233	3.994	9.838	4.059
Beta T5^a^	**8.000**	2.700	**7.964**	3.575	**8.350**	3.074	**8.729**	4.000
Beta T6	8.426	3.159	8.455	3.389	8.075	3.240	8.475	2.831
Theta F7^a^	**8.630**	2.379	**8.646**	3.221	**7.629**	2.383	**8.354**	2.353
Theta F8	7.902	2.522	8.599	3.853	7.535	2.701	8.008	2.469
Theta P3	9.203	2.812	11.351	4.238	8.845	2.311	11.263	3.820
Theta P4	9.276	2.457	11.547	4.922	8.821	2.241	10.964	3.686
Theta T5	6.105	2.015	7.182	2.841	5.931	1.821	6.865	2.256
Theta T6^a^	**6.396**	1.955	**8.340**	3.536	**5.369**	1.277	**7.450**	2.698

*M* mean, *SD* standard deviation. Benjamini–Hochberg significant results are shown in bold

^a^Main effect Condition

^b^Interaction effect Group x Condition. Based on mixed ANOVA with Group (tingles/no tingles) and Condition (Control/ASMR)

Finally it was analyzed if participants who score lower on Extraversion, Conscientiousness and participants who score higher on Openness, Neuroticism and Agreeableness are more sensitive to ASMR. This hypothesis was tested by calculating Pearson point biserial correlations between Group (tingles/no tingles) and scores on HEXACO scales. No significant correlations were found between Group (tingles/no tingles) and scores on Extraversion (*r*_*pb*(36_(38) = − 0.24, *p* = 0.14, Conscientiousness (*r*_*pb*_(38) = − 0.07 *p* = 0.69), Openness to experience (*r*_*pb*_ (38) = − 0.16, *p* = 0.34), Neuroticism (*r*_*pb*_ (38) = − 0.10, *p* = 0.51) and Agreeableness (*r*_*pb*_ (38) = 0.009, *p* = 0.96). Means of HEXACO scales are shown in Table 5.

**Table 5:** Mean scores (SD) of the HEXACO subscales for Groups (tingles n = 15; no tingles n = 23)

	Tingles	No tingles
HEXACO scale		
Openness	3.03 (0.42)	3.16 (0.41)
Conscientiousness	3.05 (0.33)	3.09 (0.26)
Extraversion	2.86 (0.18)	2.97 (0.23)
Agreeableness	3.04 (0.29)	3.03 (0.25)
Emotionality	3.09 (0.32)	3.16 (0.34)

## Discussion

The aim of the current study was to examine the effects of Autonomous Sensory Meridian Response (ASMR), in young adults, on mood, attention, heart rate, skin conductance, EEG frequency bands and the interaction with personality factors. The experience of ASMR was identified by tingling sensations while watching an ASMR video, measured by the ASMR-checklist (Barratt and Davis 2015). The effects of ASMR on the scores of the POMS, Flanker task, heart rate (HR), electrodermal activity (EDA) and EEG activity (alpha, theta, beta frequency bands at channels F7, F8, T5 and T6) were examined by presenting an ASMR video and a control video. Personality traits (neuroticism, extraversion, conscientiousness, agreeableness, openness) were measured by the HEXACO. The first expectation was that ASMR improves mood and attention. Furthermore we expected that ASMR decreases HR and increases average electrodermal activity. With respect to the EEG frequency bands we hypothesized that ASMR changes the brain activity. Finally, we hypothesized that individuals who score lower on extraversion, conscientiousness and agreeableness and individuals who score higher on openness and neuroticism are more sensitive to ASMR.

First a distinction between individuals experiencing tingles and those experiencing no tingles was made. Sensitivity to ASMR was identified by experiencing tingles during an ASMR video, because the sounds and slow movements in ASMR videos could trigger a feeling known as autonomous sensory meridian response (ASMR). In the current study 15 (37%) of 38 participants experienced tingles while watching the ASMR video. These results indicate that not everyone is sensitive to ASMR. Notably, ASMR sensitivity has been found to be associated with a decreased connectivity between the frontal lobes and sensory and attentional areas (Smith et al. 2017) and with the personality characteristics ‘increased openness to experience’ (Fredborg et al. 2017), heightened fantasizing skills (Janik McErlean and Banissy 2017) and elevated mindfulness (Fredborg et al. 2018).

The current results indicate that with respect to mood, measured by the POMS, feelings of depression decrease after watching an ASMR video in individuals who experience tingles compared to individuals who do not experience tingles. Notably, depression scores of the tingles group were not significantly lower under the ASMR condition as compared to the control condition. As a consequence, we can conclude that the ASMR video decreased depression in the tingles group only relatively to the (not significant) increase of depression in the no tingles group. Furthermore, across groups (tingles/no tingles) we found a decrease in HR while watching an ASMR video compared to watching a control video. With respect to EEG, the current results indicate a decrease of alpha power after watching an ASMR video in individuals who experience tingles compared to individuals who do not experience tingles. Besides that we found that across all participants the theta power decreases after watching an ASMR video compared to watching a control video. In addition, beta power was found to increase after watching an ASMR video. In contrast to the hypotheses, no effects of ASMR on attention and SCL were found. Furthermore, there was no significant relationship between tingles and personality traits.

With respect to mood, we expected that the scores of the POMS would change in a positive direction after watching an ASMR video compared to a control video. The results indicate that feelings of depression decrease after watching an ASMR video in individuals who experience tingles as compared to those not experiencing tingles. This is in line with the results of Barratt and Davis (2015) indicating that individuals who had been watching an ASMR video showed a better mood, and in particular a decrease of depressive mood. The present results on mood are partially comparable to the findings of Poerio et al. (2018) who used the 12-item Multi-affect Indicator measuring high activation pleasant and unpleasant affect as well as low activation pleasant and unpleasant affect. After watching ASMR videos, ASMR participants, relative to non-ASMR participants, reported increased levels of excitement and calmness, and decreased levels of stress and sadness, Particularly the ASMR-induced decreases of depressive feelings in the present study and sadness in the study of Poerio et al. seem comparable effects of ASMR videos. The increased levels of calmness and decreased levels of stress found by Poerio et al. could not be confirmed by a lower Tension score in the present study.

With respect to attention, as measured with the Flanker task, we expected that watching an ASMR video would decrease the reaction time and increase the number of correct responses on the Flanker task. The relationship between ASMR and attention has not been studied before, but improved attention might be expected as a consequence of ASMR-induced changes in the state of flow. This is an absorbing mental state of deep focus and decreased awareness of the passage of time that is often associated with optimal cognitive performance, which can be considered to be a form of attention. Participants who scored higher on flow measures experienced more triggers which are associated with ASMR content, e.g., crisp sounds, slow movements (Barratt and Davis 2015). However, the current study found no significant relationship between ASMR and performance on the Flanker task. An explanation could be that the Flanker test has a too low degree of difficulty and is therefore not sensitive enough to detect ASMR-induced cognitive improvement.

With respect to EEG, an increase of beta power in the left temporal area was found in the whole group of participants irrespective of ASMR-sensitivity after watching an ASMR video. This increase may reflect improved focused attention. In a review of Travis and Shear (2010), focused attention was characterized by beta activity which appeared to increase during meditation. This result is partly similar to the finding of increased sensorimotor rhythm (12.5–15 Hz) occurring for audio trials when comparing ASMR to control participants (Fredborg et al. 2021). However, the notion of improved focused attention seems opposite to the absence of a relationship between ASMR and performance on the Flanker task. However, the average of correct responses (i.e., > 59.5) in both groups under the ASMR and control condition is near the maximum of 60 correct responses points to a ceiling effect, which may indicate that this task was too easy and improvement of attention could have occurred with a more difficult attention task. Furthermore, Lim et al. (2019) studied the EEG activity during concentration compared to immersion. For concentration, the participants were asked to focus on a red dot at the center of a white screen, and for immersion they were asked to focus on playing a computer game. The researchers found that in the temporal lobe, the beta power decreased during concentration and increased during immersion. This result suggests that there is a relationship between increased beta power and immersion, which may be comparable with flow. As flow may be necessary to achieve feelings associated with ASMR (Barratt and Davis 2015), ASMR experience could be accompanied with an increase in beta power.

With respect to the physiological effects of ASMR, the current results indicate a HR decrease while watching an ASMR video in all participants, disregarding having tingles or not. This finding is partly in line with the results of Poerio et al. (2018) who found that HR decreased in non-ASMR responders when watching ASMR videos, although a more pronounced decrease in ASMR responders was seen. This finding suggests that ASMR videos may be relaxing even in not ASMR-sensitive individuals and explains why these individuals report benefits from watching an ASMR video even if they are unable to experience tingles. This suggestion is supported by the (nearly significant) relationship between HR and Tension scores for all participants only under the ASMR condition. As heart rate variability (HRV) has been found to be higher under stress (Kim et al. 2018) and ASMR video’s may be relaxing, watching an ASMR video can be expected to decrease HRV. A more stable HR increases the likelihood to reliably relate individual HR with Tension score. Notably, due to trigger preferences and characteristics of experimental setup some of the participants within the no tingles group might yet have been ASMR-sensitive not achieving tingles but only relaxation. This idea would be in agreement with previous findings that ASMR-experiencers only reported tingles during 5.9% of a 7 min testing session, while relaxation without tingles occurred during 51.4% of the testing session (Lochte et al. 2018).

No significant effects of ASMR were found for the skin conductance registrations. An explanation for the absence of a significant change in average electrodermal activity during the ASMR video could be the smaller number of participants who experienced tingles (*n* = 15) in the current study compared to that (*n* = 55) in the study of Poerio et al. (2018). It might be that the EDA effect is harder to detect because it is mainly associated with ASMR tingling and not with the overall relaxation effect that ASMR has. Therefore, it can be recommended that EDA should specifically be examined during tingling sensations. Another explanation can be supplied by the present findings that ASMR individuals with low scores on Conscientiousness showed a (marginally significant) reduction of HR, relative to the no tingles group, and a marginal significant increase in EDA. Lower scores on conscientiousness have been associated with higher reactivity to daily stressors and higher nervousness (Komulainen et al. 2014). As a consequence, only that part of our group of ASMR individuals that scored lower on Conscientiousness exhibits to the ASMR-induced physiological changes. As mentioned above, individuals experiencing ASMR have been found to score lower on scales of Conscientiousness (Fredborg et al. 2017; Janik McErlean and Banissy 2017; Roberts et al. 2021). It may well be true that individuals scoring low on conscientiousness experience tingles and those experiencing tingles with still lower scores on conscientiousness exhibit physiological reactivity to ASMR videos. The present findings of decreased HR and increased EDA observed in ‘low conscientious’ individuals are in line with previously reported ASMR- induced decrease of HR and increase of SCL in ASMR individuals (Poerio et al. 2018).

With respect to the EEG, in addition to the above-mentioned increase of beta power the current results indicate a decrease of alpha power in the right temporal and parietal area while watching an ASMR video in individuals who experience tingles. This is opposite to the findings of Fredborg et al. (2021) who found increased alpha activity in frontal and parietal brain regions in ASMR responders. Notably, they found a decrease in alpha power in control subjects. The authors suggested that individuals who do not experience ASMR might perceive ASMR stimuli as strange or even make feel them embarrassed. A similar explanation could be that our participants experienced tingles as aversive, which may explain the decrease of alpha activity. However, it appeared that only one participant experienced watching the video as annoying, which makes this explanation unlikely.

Furthermore, a decrease of theta power in the left frontal and right temporal areas was found in the whole group of participants while watching an ASMR video. The present findings of increase of beta power and decrease of alpha and theta power are thus opposed to the expectation that watching an ASMR video is related to relaxation and therefore associated with increase of alpha or theta power. On the contrary, the present findings of a decrease in alpha and theta power and an increase in beta power suggest that ASMR stimulation is mainly characterized by focused attention and immersion (Lim et al. 2019; Travis and Shear 2010). This idea is further supported by previous findings of elevated absorption in ASMR-experiencers, suggesting that the ability to get deeply immersed with the current experience substantially contributes to the experience of ASMR (Janik McErlean and Osborne-Ford 2020; Roberts et al. 2019).

As is mentioned in the introduction, Poerio et al. (2018) found that ASMR induces relaxation as indicated by a reduced HR as well as excitement as indicated by self-ratings and increased SCL in individuals who experience ASMR. In the present study we found similar results for HR and EDA in ‘low conscientious’ participants. Moreover, we found brain activation and an fMRI study by Lochte et al. (2018) in ASMR individuals showed increased activation in regions associated with emotional arousal (dACC and Insula/IFG). In addition, as tingling sensations are associated with increases in pupil diameter, tingles themselves seem the reflection of increased arousal. The complex relationship of ASMR with relaxation and arousal can be explained by the concept of directional fractionation, as was examined in a study predicting that attention to the environment leads to sympathetic-like pupil dilatation and parasympathetic-like cardiac slowing (Libby et al. 1973). Pupillary diameters and heart rates were recorded in 34 males who watched 30 pictures of different attractivity. Pupillary dilatation and cardiac slowing increased as the Attention-Interest value of the pictures increased. The decreased HR and increased SCL as well as the occurrence of tingles and pupil diameter increase in ASMR may be an instance of directional fractionation. In addition, the sympathetic arousal indicated by tingles and pupil dilation may induce flow following an inverted u-curve characterized by moderate physiological arousal facilitating flow-experience (Peifer et al. 2014).

Finally, no evidence was found for the hypothesis that individuals who are more sensitive to ASMR, as defined by experiencing tingles, score higher on Openness-to-Experience and lower on Conscientiousness (Fredborg et al. 2017; Janik McErlean and Banissy 2017), nor with the reported higher Neuroticism and lower Agreeableness (Fredborg et al. 2017). One-tailed tests only indicated a trend (*p* = 0.069) of lower extraversion scores in ASMR sensitive individuals. It must be noted that the HEXACO-SPI is not identical to the BFI as used by the previous cited studies. Although most HEXACO and BFI scales correlate well as is mentioned under the section Personality and means of the HEXACO scales as shown in Table 5 are quite similar to those reported by Fredborg et al. (2017) and Roberts et al. (2021), the use of a different questionnaire may have caused some discrepancy in results. An additional explanation for the lack of significant results in the present study could be that the sample sizes of participants who experienced ASMR in the cited studies (*n* = 284, *n* = 83 and *n* = 146, respectively) were substantially larger than that of the present study (*n* = 15).

### Limitations

One limitation of the present study is the use of only one stimulus video. Presenting this particular ASMR video might have excluded ASMR-sensitive individuals who did not respond with tingles to the present stimulus selection. As is mentioned in the introduction ASMR trigger preferences vary across audio -, visual -, or tactile stimuli such as whispering, soft-speaking, tapping, scratching, slow and expert hand movements and close personal attention (Barratt and Davis 2015). Secondly, in the present study the occurrence of tingles was the dividing criterion. We decided to make the distinction according to this sole criterion, as the perception of tingles is quite straightforward and one of the most important components of the ASMR experience. Notably, tingling sensations have been found to be accompanied with increases in pupil diameter. This indicates that they have a physiological basis and may be considered to be the core of the experience itself (Valtakari et al. 2019). The present limitations can be avoided by presenting a more diverse selection of ASMR triggers and by dividing the group of participants according to a broader selection of components of the ASMR experience.

In addition, the lack of baseline measurements and the use of only one video per condition does not allow for revealing potential differences in engagement or arousal elicited by the videos. As EDA, EEG and HR measures are sensitive to general arousal states, differences between conditions may be due to specific properties of the videos. However, as decreased alpha power under the ASMR condition was only found in participants experiencing tingles, this effect can be considered to be part of the ASMR experience. To clarify possible confounding effects of differences in video characteristics, future studies should include baseline measurements and measures of the engagement of participants with the stimuli.

## Conclusions

From the present results we may conclude that ASMR decreases feelings of depression in individuals who are sensitive to ASMR. Moreover, ASMR videos reduce heartrate in individuals, irrespective of being sensitive to ASMR or not. In addition, the EEG recordings of the present study show that ASMR videos decrease alpha and theta power and increases beta power, which may contribute to the idea that ASMR is related to arousal and a form of focused attention, like ‘state of flow’ or absorption.

## Data Availability

The datasets generated during and/or analyzed during the current study are available from the corresponding author on reasonable request.
